# Gut microbiota remodeling and immune gene modulation by *Semiliquidambar cathayensis* protect *Misgurnus anguillicaudatus* against *Aeromonas hydrophila*: integrative transcriptomic and 16S rRNA analysis

**DOI:** 10.3389/fimmu.2026.1949998

**Published:** 2026-09-16

**Authors:** Zepu Wang, Zihan Sun, Mingjie Deng, Yi Shen

**Affiliations:** 1 Department of Cardiothoracic Surgery, Nanjing Jinling Hospital, Affiliated Hospital of Medical School, Nanjing University, Nanjing, China; 2 Institute of Esophageal Cancer, Medical School, Nanjing University, Nanjing, China; 3 School of Traditional Chinese Medicine, Wenzhou Medical University, Wenzhou, China; 4 School of Pharmaceutical Sciences, Wenzhou Medical University, Wenzhou, China

**Keywords:** 16S rRNA sequencing, Aeromonas hydrophila, Misgurnus anguillicaudatus, Semiliquidambar cathayensis, transcriptomics

## Abstract

This study aims to explore the regulatory mechanism of *Semiliquidambar cathayensis* extract on the immune stress induced by *Aeromonas hydrophila* in *Misgurnus anguillicaudatus*. Through transcriptomics and 16S rRNA sequencing techniques, the changes in gene expression profiles of *M. anguillicaudatus* muscle tissues and the structure of the intestinal microbiota were analyzed separately. The transcriptomic results showed that a total of 1,080 differentially expressed genes were screened out after *S. cathayensis* treatment, among which 509 were upregulated and 571 were downregulated. GO enrichment analysis indicated that these genes mainly participated in cellular processes, biological regulation, and stress responses. KEGG enrichment analysis revealed that they were significantly enriched in immune-related pathways such as antigen processing and presentation, complement and coagulation cascade reaction, and phagosome. The 16S rRNA sequencing results showed that *S. cathayensis* treatment significantly reshaped the intestinal microbiota structure. The dominant bacterial genera changed from Lactococcus and Myroides to Elizabethkingia, with Elizabethkingia meningoseptica accounting for an absolute majority in the treatment group, with an abundance of up to 61.70%. The integrated analysis indicated that *S. cathayensis* may affect the expression of host immune-related genes by regulating the abundance of specific bacterial communities (especially the Elizabethkingia genus), thereby alleviating the immune stress induced by *A. hydrophila*. This study provides a theoretical basis for the development and application of *S. cathayensis* as an aquatic immune enhancer, and also offers a new research perspective for exploring the immune regulatory effects of traditional Chinese medicine through ‘microbiota-host’ interaction.

## 1 Introduction

The *Misgurnus anguillicaudatus* is an important native freshwater economic fish species, widely distributed in China, Japan, South Korea, and other countries in Southeast Asia (1, 2). Its edible proportion is as high as 80%, significantly higher than that of ordinary freshwater fish (3). The meat is tender, the flavor is delicious, and the nutritional value is outstanding (4). *Aeromonas hydrophila*, as a common opportunistic pathogen in aquaculture, is one of the main pathogens causing bacterial septicemia, ulcer disease, and intestinal inflammation in freshwater fish species such as *M. anguillicaudatus (*5, 6). This bacterium damages the host’s intestinal barrier by secreting exotoxins, adhesion factors, and forming biofilms, activates the Toll-like receptor (TLR) signaling pathway, induces a strong inflammatory response, and in severe cases, can lead to large-scale death of farmed animals (7). The *M. anguillicaudatus* is an important freshwater economic aquaculture species in China (8). In the intensive aquaculture mode, *A. hydrophila* diseases occur frequently, becoming a key factor restricting the development of the industry. Therefore, developing safe and efficient immune enhancers to prevent *A. hydrophila* infection has significant practical significance in production (9, 10).

In recent years, Chinese herbal medicine has attracted much attention in the field of aquatic animal immune enhancers due to its abundant resources, multi-target regulation and low residue characteristics (11, 12). *Semiliquidambar cathayensis*, as a traditional medicinal plant in China, has its roots, stems and leaves rich in flavonoids, phenolic acids and triterpenoids (13, 14). Modern pharmacological studies have shown that the extract of *S. cathayensis* exhibits significant anti-inflammatory, antioxidant and immune regulatory activities in mammalian models, and can alleviate inflammatory responses by inhibiting the nuclear factor κB (NF-κB) and mitogen-activated protein kinase (MAPK) signaling pathways (15). However, there is currently a lack of research on the immune regulatory mechanism of *S. cathayensis* in aquatic animals, especially in mudfish, and its protective effect and potential targets in the Pseudomonas aeruginosa infection model have not been clarified.

It is worth noting that the intestine is not only the primary site for digestion and absorption in aquatic animals, but also the largest immune organ of the body (16, 17). The intestinal mucosal immune system, together with the resident intestinal microbial community, forms a complex ‘microbiota-host’ interaction network (18). A stable intestinal microbiota can maintain the integrity of the intestinal barrier and regulate the function of immune cells through competitive exclusion and the secretion of metabolites such as short-chain fatty acids; while pathogen infections often lead to intestinal microbiota dysbiosis, manifested as an increase in the abundance of opportunistic pathogens and a decrease in the abundance of beneficial bacteria, further exacerbating local and even systemic inflammation (19). Therefore, restoring the balance of intestinal microecology is regarded as one of the important mechanisms by which traditional Chinese medicine exerts immunomodulatory effects. However, existing studies are mostly limited to single-level descriptions, that is, only through 16S rRNA sequencing to describe changes in the microbiota structure, or only through transcriptome analysis of host gene expression, with few conducting correlation analyses of the two and systematically revealing the mechanism of action of traditional Chinese medicine from the perspective of ‘Chinese medicine - microbiota - host’ interaction.

By integrating 16S rRNA high-throughput sequencing technology and transcriptome sequencing technology, the diversity and community structure of the intestinal microbiota of loaches (*M. anguillicaudatus)*, as well as the changes in gene expression profiles in immune-related tissues were analyzed separately. Through Spearman correlation analysis, an association network between differentially abundant bacterial genera and key immune genes was constructed, aiming to reveal the potential mechanism of *S. cathayensis* alleviating immune damage induced by *A. hydrophila* in loaches at the multi-omics level. The research results not only provide a theoretical basis for the development and application of *S. cathayensis* as an aquatic immune enhancer, but also offer a new perspective for exploring the anti-infection effect of traditional Chinese medicine through regulating the interaction between intestinal microbiota and host immunity.

## 2 Materials and methods

### 2.1 Materials

The dried stem bark of *S. cathayensis* was purchased from the medicinal market in Jinxiu Yao Autonomous County, Guangxi Province. 500 g of the crude powder of the dried stem bark of *S. cathayensis* was taken and extracted twice with 8 times the volume of 95% ethanol, each extraction lasting for 2 hours. The filtrates were combined, evaporated under reduced pressure until no alcohol smell remained, and the total extract was obtained. The total extract was dispersed in an appropriate amount of distilled water, and then extracted with equal volumes of petroleum ether three times. The petroleum ether phases were combined, the solvent was evaporated under reduced pressure at 40 °C, and the resulting oil extract was freeze-dried and stored in a sealed, dark container at 4 °C for future use.

### 2.2 Experimental animal and treatment

The *M. anguillicaudatus* used in the experiment were purchased from a certain aquatic breeding base in Zhejiang Province. After arriving at the laboratory, all individuals were temporarily kept in aerated tap water for 15 days to adapt to the environmental conditions. After the temporary keeping period, 40 healthy *M. anguillicaudatus* with normal body color and approximately the same size were selected for the subsequent experiments. The average body length of the *M. anguillicaudatus* was 12 cm. The experimental aquaria were of dimensions 37 cm in length, 31.5 cm in width, and 13.5 cm in height.

The 40 *M. anguillicaudatus* were randomly divided into two groups: the control group (CK1) and the *S. cathayensis* extract treatment group (B2). The *A. hydrophila* was inoculated into LB liquid medium and cultured at 28 °C for 12 hours. The bacterial cells were centrifuged and resuspended in sterile phosphate-buffered saline (PBS, Biosharp Biotechnology Co., Ltd.) and adjusted to a concentration of 1×10^6^ CFU/mL. 100 μL of the bacterial suspension was injected into the dorsal muscle of each mudfish for infection. After infection, the treatment group was fed with feed containing the *S. cathayensis* oil ether extract, while the control group was fed with the basic feed. 24 hours after infection, the *M. anguillicaudatus* were anesthetized and sacrificed using MS-222 (Beijing Greenhengxing Biotechnology Co., Ltd.). For anesthesia, loaches were immersed in a solution of 140 mg/L MS-222 (tricaine methanesulfonate) until equilibrium loss and a complete loss of opercular movement were observed. Subsequently, for euthanasia, the anesthetic concentration was increased to 300 mg/L, and loaches were maintained in this solution for an additional 10 minutes to ensure deep anesthesia and death, followed by decapitation to confirm death. The body surface was first wiped with 75% ethanol for disinfection, then the dorsal muscle tissue was cut under sterile conditions, placed in an enzyme-free centrifuge tube, immediately frozen in dry ice, and finally transferred to a -80 °C ultra-low temperature refrigerator for subsequent RNA extraction and transcriptome sequencing analysis. At the same time, the intestinal contents were collected under sterile conditions for 16S rRNA sequencing. All animal experiment operations complied with the relevant requirements of animal ethics.

### 2.3 RNA extraction, library construction and sequencing

The total RNA from the *M. anguillicaudatus* muscle tissue was extracted using the TRIzol method (Life Technologies), and the operation steps were carried out strictly in accordance with the instructions of the kit. The extracted RNA was then subjected to quality assessment through agarose gel electrophoresis, Nanodrop microspectrophotometer, and Agilent 2100 bioanalyzer, to evaluate its integrity, purity, and concentration.

After passing the quality inspection, magnetic beads labeled with Oligo (dT) were used to enrich the mRNA in the total RNA that carried the polyA tail. The enriched mRNA was subjected to ultrasonic fragmentation, with the fragment length controlled at approximately 300 bp. This length effectively balances the ratio of the adapter sequence to the efficiency of sequencing clusters. The fragmented mRNA was used as a template to synthesize the first strand of cDNA using random primers and M-MuLV reverse transcriptase. Subsequently, RNA was degraded using RNaseH, and the second strand of cDNA was synthesized using dNTPs in the DNA polymerase I system. The double-stranded cDNA was then end-repaired, modified, and ligated with adapters, and cDNA fragments of approximately 200 bp were selected using AMPure XP magnetic beads. After PCR amplification and purification, the library construction was completed.

After library construction, quality inspection was conducted using the Agilent 2100 bioanalyzer, and the total concentration and effective concentration of the library were determined. Based on the effective concentration and target data volume, libraries with different Index labels were mixed in proportion, diluted to 2 nM, and subjected to denaturation treatment to form single-stranded libraries. Finally, based on the Illumina sequencing platform, high-throughput sequencing was performed on the above libraries using the dual-end sequencing mode.

### 2.4 Bioinformatics analysis

The raw sequencing data were subjected to quality control processing, with the removal of reads containing adapter sequences, those shorter than 50 bp in length, and those with an average quality value lower than Q20. Eventually, high-quality and valid sequences (Clean reads) were obtained. These sequences were then spliced from scratch to generate transcript sequences. The longest transcript was selected as the Unigene after clustering. Based on the Unigene sequences, the Blast comparison tool was used to compare with public databases such as NR, SwissProt, KEGG, eggNOG, GO, and Pfam to complete the functional annotation of the genes. At the same time, open reading frame (ORF) and simple repeat sequence (SSR) prediction analyses were conducted. Additionally, the quality-controlled sequences were compared back to the Unigene to obtain the Read Count values corresponding to each Unigene, and further differential expression analysis, enrichment analysis, and variant detection were carried out based on this.

### 2.5 Real-time fluorescent quantitative PCR verification

To verify the reliability of the transcriptome sequencing results, some differentially expressed genes were selected for real-time fluorescence quantitative PCR (qRT-PCR) analysis (20). The total RNA from liver tissues was reverse-transcribed into the first strand cDNA using the TransGen Biotech universal cDNA synthesis super mixture (China). This cDNA was used as the template for qPCR amplification. β-actin was selected as the internal reference gene, and specific primers for the target genes and the internal reference gene were designed using the Primer v5.0 software. The primer sequences are shown in **Table 1**. The qPCR reaction was carried out using the Green qPCR super mixture (TransGen Biotech, China). The reaction program was as follows: 30 s at 95 °C for pre-denaturation; then 5 s at 94 °C for denaturation, 15 s at 55 °C for annealing, and 10 s at 72 °C for extension, for a total of 40 cycles. Three biological replicates were set for each sample. The relative expression levels of each target gene were calculated using the 2^-ΔΔCt^ method.

**Table 1:** The genes and primers used for qRT-PCR analysis.

Gene name	Forward primer (5′–3′)	Reverse primer (5′–3′)	Product size (bp)
thrsp	CTGCGAGGCTGCCGAAAACA	TACAGGGCTGCGTGACTCTTACC	129
hexokinase 4	ACTCGCTGGCGTCATCAACCTAAT	CACCCCGACCACTCCCTTCTTC	191
radil2a	GGGCTTTCCGGTTGGGTTACTGAT	CTGTCTGACTCCGCTGCTCTCCAC	139
pglyrp6	CGCCGCACCCCGTCTCATA	AAAGCCAAGTAGTTACCAGCATA	192
delta-9-desaturase	GAACCGACCGTACGACAGCAACAT	GGCCAGACCCAACAAACACATCA	184
pcsk2	AAGGGCCGAGGTGGAAAGGGAAGC	CTGAAGGTGGAGGCGAGCGTGGAC	121
LOC129448634	TAAGCCTTCAGGTGGGGATAAA	CAGCAAGGCCAAGAGGTAAAAA	164
tgfbr2l	AGCGGGGTGGCACTTCTCAG	CGATGGGCGATTGGCACCTT	199
ppp3cca	CAGCACTGCGCATCATCAAC	CCACGTAGTCGCCAAGGAAAAG	183
egr1	AAAAAGGCGGAGAAAGGCGTCGTG	CCGGGGAGGGGTAGGATGTGATTG	109
vdac1	CCGCCAGGGACATTTTCACT	AGCGTTCCTGCCACCTTACTG	136
fosab	AGAAATCCGCCCTACAAAATGACA	AGGCGACGGGAGAAACGGACTG	147
β-actin	AGAGAGAAATTGTCCGTGAC	GCCAATGGTGATGACCTGT	140

### 2.6 DNA extraction detection and 16S rRNA sequencing

After thawing the intestinal samples, accurately weigh 0.2 to 0.5 g and place it in a centrifuge tube containing lysis solution. Homogenize the sample using a grinding instrument at a frequency of 60 Hz. The total DNA of the sample is extracted using the OMEGA Soil DNA Kit (D5635-02) (Omega Bio-Tek, Norcross, GA, USA), following the instructions of the kit. The extracted DNA is evaluated for fragment integrity by 0.8% agarose gel electrophoresis and its concentration and purity are determined using a Nanodrop micro spectrophotometer. Using the extracted DNA as the template, the bacterial 16S rRNA gene V3-V4 hypervariable region is selected as the amplification target. The primers are 338 F (5′-ACTCCTACGGGAGGCAGCAG-3′) and 806 R (5′-GGACTACHVGGGTWTCTAAT-3′). The amplification system uses NEB Q5 high-fidelity DNA polymerase. The reaction procedure is as follows: 5 min of pre-denaturation at 98 °C; 30 s of denaturation at 98 °C, 30 s of annealing at 52 °C, and 45 s of extension at 72 °C, for a total of 25 cycles; finally, 5 min of final extension at 72 °C. The amplification products are detected by 2% agarose gel electrophoresis, and then purified using the AxyPrep DNA gel recovery kit (Aixin Biotechnology, USA). The purified products are quantified using the Quant-iT PicoGreen double-stranded DNA quantitative kit (Thermo Fisher, USA) on the FLx800 microplate reader (Berten, USA).

The fungal project uses the ITS1 region as the amplification target, with the primer sequences being ITS5 (5′-GGAAGTAAAAGTCGTAACAAGG-3′) and ITS2 (5′-GCTGCGTTCTTCATCGATGC-3′). The archaeal project selects the V4-V5 region of the 16S rRNA gene, with the primers being 524 F (5′-TGYCAGCCGCCGCGGTAA-3′) and 958 R (5′-YCCGGCGTTGAVTCCAATT-3′). The 5′ end of the former primers all carry 7–10 bp barcode sequences, which are used to distinguish different samples within the same library. PCR amplification uses NEB Q5 high-fidelity DNA polymerase, and the reaction system and conditions are the same as those for the bacterial project. The amplification products are quantified using the Quant-iT PicoGreen dsDNA quantitative kit on a Microplate reader (BioTek, FLx800), and then mixed in proportion to the required data volume for each sample.

After mixing the amplification products of each sample at the same molar concentration, a sequencing library is constructed and sequenced on the Illumina NovaSeq platform with 2×250 bp paired-end sequencing. The original sequences are filtered through quality control and decontamination, etc., and then clustered using QIIME2 software based on the Silva database for species annotation. Subsequently, the diversity of the microbiota and the differences in community structure are analyzed. The 16S rRNA sequencing work was completed by Shanghai Pansenuo Biotechnology Co., Ltd.

### 2.7 16S rRNA sequencing data analysis

The 16S rRNA sequencing data were analyzed bioinformatically using the QIIME2 software. The original sequences were decoded using the demux plugin, and the primer sequences were removed using the cutadapt plugin. Then, the sequences were subjected to quality filtering, denoising, assembly, and removal of chimeras using the DADA2 method to obtain high-quality sequences (21). DADA2 performed deduplication and merging with a 100% similarity threshold, generating amplicon sequence variants and their abundance tables. The ASV characteristic sequences were compared with the Greengenes database to obtain the taxonomic information of each ASV, and rare ASVs with an abundance lower than 0.001% of the total sequencing quantity of all samples were removed.

Based on the obtained ASV abundance matrix, Alpha diversity indices (Chao1, Shannon, Simpson) were calculated using QIIME2 and diluted curves were plotted; Beta diversity analysis was performed based on the Bray-Curtis distance, and visualization was achieved through principal coordinate analysis. The SparCC method was used to construct a microbial association network, with a pseudocount value of 10^-6^. The RMThreshold package in R language was used to determine the correlation coefficient threshold and significance based on the random matrix theory, and the network was visualized using the ggraph package. Using the PICRUSt2 software, the microbial community metabolic functions were predicted based on the MetaCyc and KEGG databases, and the inter-group differential metabolic pathways were selected based on the corrected P value and fold difference.

### 2.8 Experiments related to physiological indicators

Bacterial Challenge and Sample Collection: Both the control (CK1) and treatment (B2) groups were challenged by immersion in water containing an *A. hydrophila* suspension (1×10^5^ CFU/mL). Post-challenge, all environmental and feeding regimens remained identical to the pre-challenge period. Liver tissues were aseptically collected from three *M. anguillicaudatus* per group at each of the following time points: 3, 6, 12, 24, 48, and 72 hours post-infection. Tissues from each triplicate were pooled to form a biological replicate, immediately snap-frozen in liquid nitrogen, and stored at -80 °C until analysis.

Hepatic antioxidant and inflammatory indices were determined as follows. Frozen liver tissues were homogenized in ice-cold physiological saline or an appropriate buffer solution. The supernatant was collected after centrifugation and used for the measurement of enzyme activities and metabolite concentrations. The activities of critical antioxidant enzymes, including superoxide dismutase (SOD), catalase (CAT), and glutathione peroxidase (GPx), as well as myeloperoxidase (MPO) activity, a hallmark of inflammation, were assayed. Furthermore, the level of malondialdehyde (MDA), a major product of lipid peroxidation, was quantified. All colorimetric and spectrophotometric detections were performed using commercial diagnostic kits purchased from Nanjing Jiancheng Bioengineering Institute (Nanjing, China), strictly following the manufacturer’s instructions.

## 3 Results

### 3.1 Quality control of sequencing data and overview

By sequencing the control group (CK1 group) and the *S. cathayensis* treatment group (B2 group), 42,743,116 and 50,832,142 raw reads were obtained respectively. After quality control and filtering of low-quality data using the fastp software, 41,961,622 (98.17%) and 49,892,946 (98.15%) high-quality reads were obtained respectively. The more balanced the base composition, the higher the quality, and the more accurate the subsequent analysis. The base composition and quality distribution of the high-quality reads were analyzed, and the results showed that the GC content of the CK1 group and the B2 group was 41.01% and 41.47% respectively, and Q20 (base recognition accuracy ≥ 99%) was 97.59% and 97.55% respectively, and Q30 (base recognition accuracy ≥ 99.9%) was 95.72% and 95.63% (**Table 2**).

**Table 2:** Summary of the sequence analyses.

Sample	Raw reads	Clean reads (%)	Q20 (%)	Q30 (%)	GC ( %)
CK1	42743116	41961622 (98.17)	97.59	95.72	41.01
B2	50832142	49892946 (98.15)	97.55	95.63	41.47

Raw reads: From whole-genome sequencing. Clean reads: The number of reads after removing low-quality sequences. The subsequent analysis is based on clean reads.Q20 and Q30: The percentage of bases with Phred values >20 and >30, respectively. GC: The GC proportion of filtered sequence bases.

Sequence assembly was performed using the Trinity software, and the assembly quality was evaluated by the N50 value. The larger the N50 value and the fewer the number, the better the assembly quality. All Unigenes were sorted by length from long to short and cumulated successively. When the cumulative length reached 50% of the total length, the corresponding fragment length and quantity were the length and quantity of the Unigene N50. In this study, the N50 quantity was 11,721 and the length was 1,939 bp; the maximum length of Unigenes was 21,084 bp, the minimum length was 309 bp, and the average length was 1,099 bp (**Table 3**). A total of 82,932,484 Unigenes were obtained, with lengths ranging from 309 bp to 21,084 bp, mainly concentrated in the 300–600 bp range, and the distribution of other length ranges was relatively small (**Figure 1**).

**Table 3:** Assembly quality statistics.

N50 number	N50 length	Max length	Min length	Average length
11721	1939	21084	309	1099

N50 number: the number of Unigene N50. N50 length: The length of Unigene N50. Max length: Assemble the longest unigene length. Min length:Assemble the shortest unigene length. Average length: The average length of unigene is assembled.

**Figure 1:**
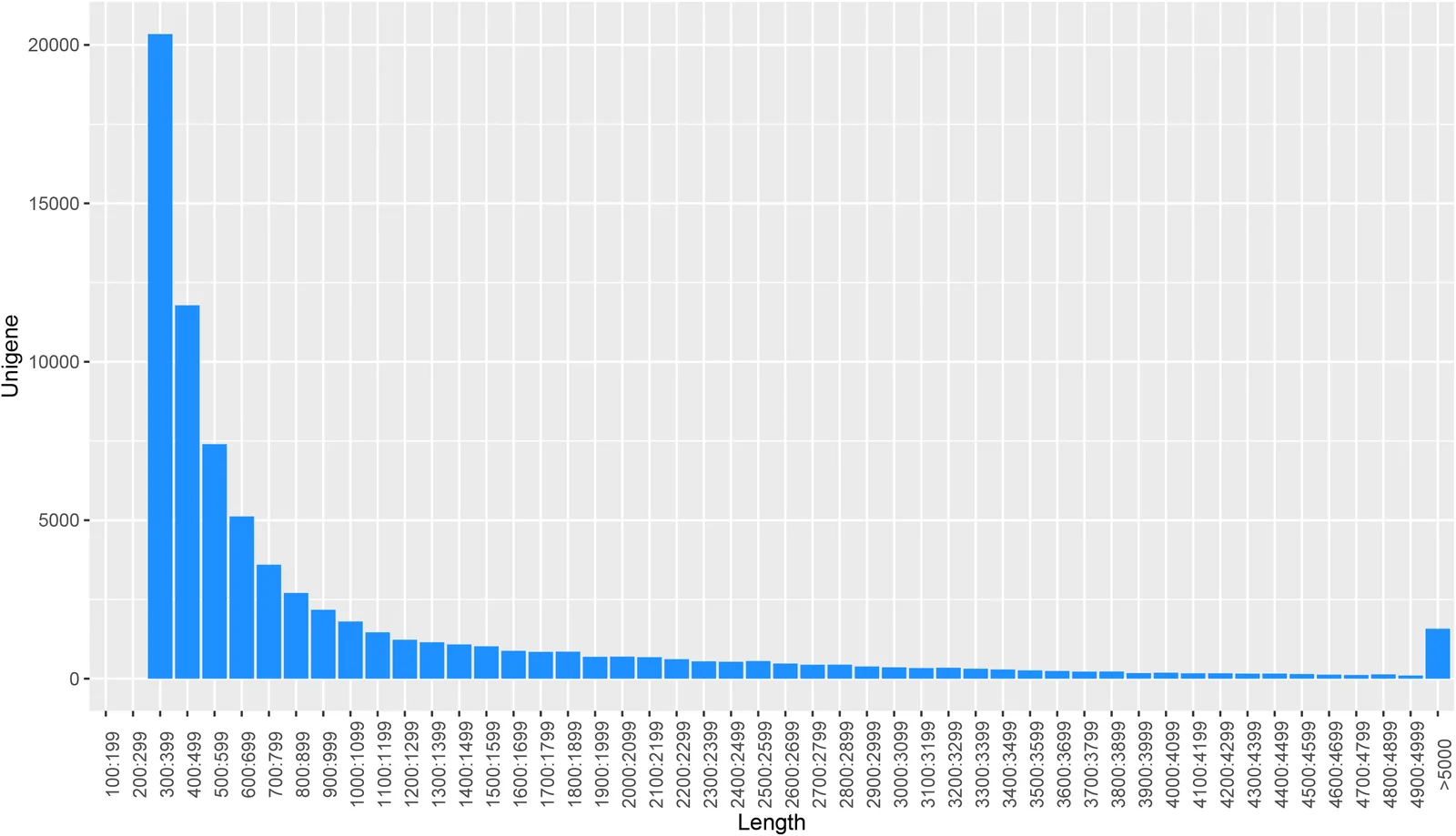
Length distribution of Unigene. The abscissa indicates the length of assembled Unigenes, and the ordinate indicates the number of Unigenes under the corresponding length.

### 3.2 Perform gene function annotation for Unigene

To assess the functions of the obtained Unigenes, their gene functions were annotated. The Unigene sequences were compared to the NR (non-redundant protein sequence database), SwissProt, KEGG (Kyoto Encyclopedia of Genes and Genomes), and eggNOG (direct homologous protein group alignment) databases using Blast. The results showed that among the 75,430 Unigene sequences, a total of 10,188 sequences were annotated in all the databases. Regarding the annotation status of each database, the NR database had the highest annotation rate (39.87%), followed by the eggNOG database (38.10%), the SwissProt database (29.06%), and the KEGG database (20.83%) (**Table 4**).

**Table 4:** Annotation statistics of four major databases.

Database	Number	Percentage (%)
NR	30070	39.87
GO	20184	26.76
KEGG	15709	20.83
Pfam	18442	24.45
eggNOG	28742	38.10
Swissprot	21916	29.06
In all database	10188	13.51

Database: Type of database. Number: Represents the number of unigenes successfully annotated in the database. Percentage (%): represents the proportion of unigenes successfully annotated in the database to the total unigenes. In all database: The number of Unigenes annotated in all databases.

#### 3.2.1 NR annotation

The Unigene sequences were aligned against the NR (Non-Redundant Protein Sequence) database to analyze the distribution of e-values, sequence similarity, and species composition. A lower e-value indicates a more reliable alignment. Analysis of the e-value distribution revealed that sequences with e-values less than 1e-15 accounted for over 70% of total alignments, indicating high confidence in the annotation results. Sequence identity reflects the homology between Unigenes and known sequences. The statistical results showed that the highest proportion of sequences (42.63%) fell within the 60%–80% identity range, followed by the 40%–60% range (31.05%), suggesting that the majority of Unigenes exhibit moderate to high homology with known sequences. For each Unigene, the sequence with the best alignment result (i.e., the smallest e-value; the first was selected in cases of ties) was considered the homologous sequence, and its corresponding species was determined. The number of homologous sequences for each species was then calculated. The results showed that the highest proportion of homologous sequences matched *Pimephales promelas* (fathead minnow), accounting for 34.29% of the total, followed by sequences assigned to other species (23.07%). The remaining sequences were distributed among the following species: *Anabarilius grahami* (8.92%), *Sinocyclocheilus rhinocerous* (7.91%), *Carassius auratus* (6.95%), *Cyprinus carpio* (6.48%), *Onychostoma macrolepis* (6.20%), and *Triplophysa tibetana* (6.18%) (**Figure 2**).

**Figure 2:**
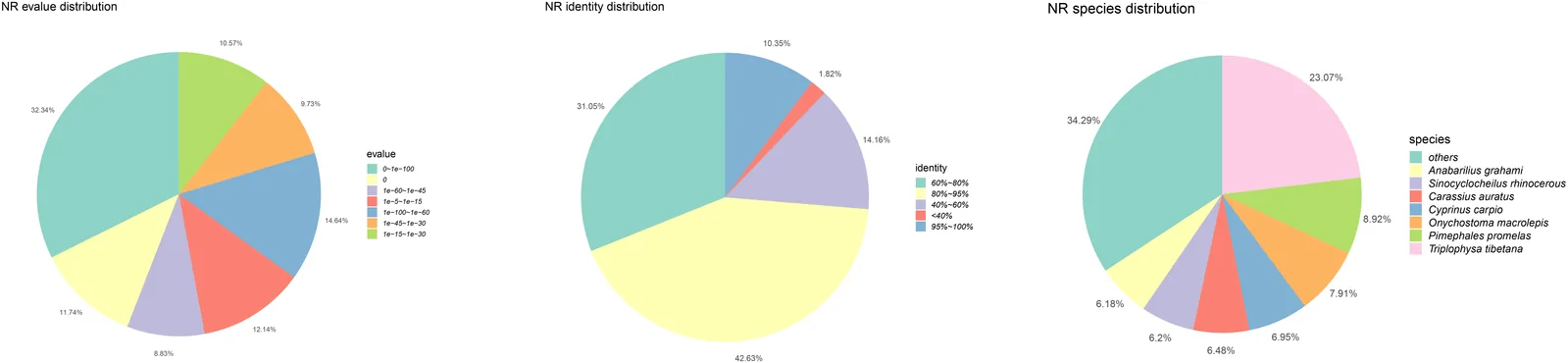
NR annotation statistics.

#### 3.2.2 KEGG annotation

KEGG (Kyoto Encyclopedia of Genes and Genomes) is a database that integrates genomic, chemical, and system functional information, aiming to reveal the genetic material and metabolic blueprint of life phenomena. The KEGG annotation results (**Figure 3**) show that a total of 19,117 Unigene were annotated to 35 pathways. In the metabolism category, the pathway with the largest number of annotations was lipid metabolism (519), followed by carbohydrate metabolism (484) and amino acid metabolism (431); the pathway with the fewest annotations was the metabolism of terpenoids and polyketides (49) and the biosynthesis of other secondary metabolites (65). In the genetic information processing category, the folding, sorting, and degradation pathway had the largest number of annotations (607), followed by translation (463); the number of annotations in information processing-related pathways was relatively small. In the environmental information processing category, the signal transduction pathway had the largest number of annotations (2,793), accounting for 70.8% of the total annotations in this category; the signal molecules and interaction pathway (1,060) and membrane transport pathway (93) had relatively fewer annotations. In the cellular processes category, the transport and catabolism pathway had the largest number of annotations (1,244), followed by cell growth and death (991) and cellular community - eukaryotes (985), while the cellular community - prokaryotes pathway had the fewest annotations (24). In the organismal systems category, the immune system (1,756) and endocrine system (1,454) had the largest number of annotations, accounting for 24.5% and 20.3% of the total annotations in this category respectively; pathways such as digestive system (760), development and regeneration (645), and environmental adaptation (521) also had relatively high annotation quantities.

**Figure 3:**
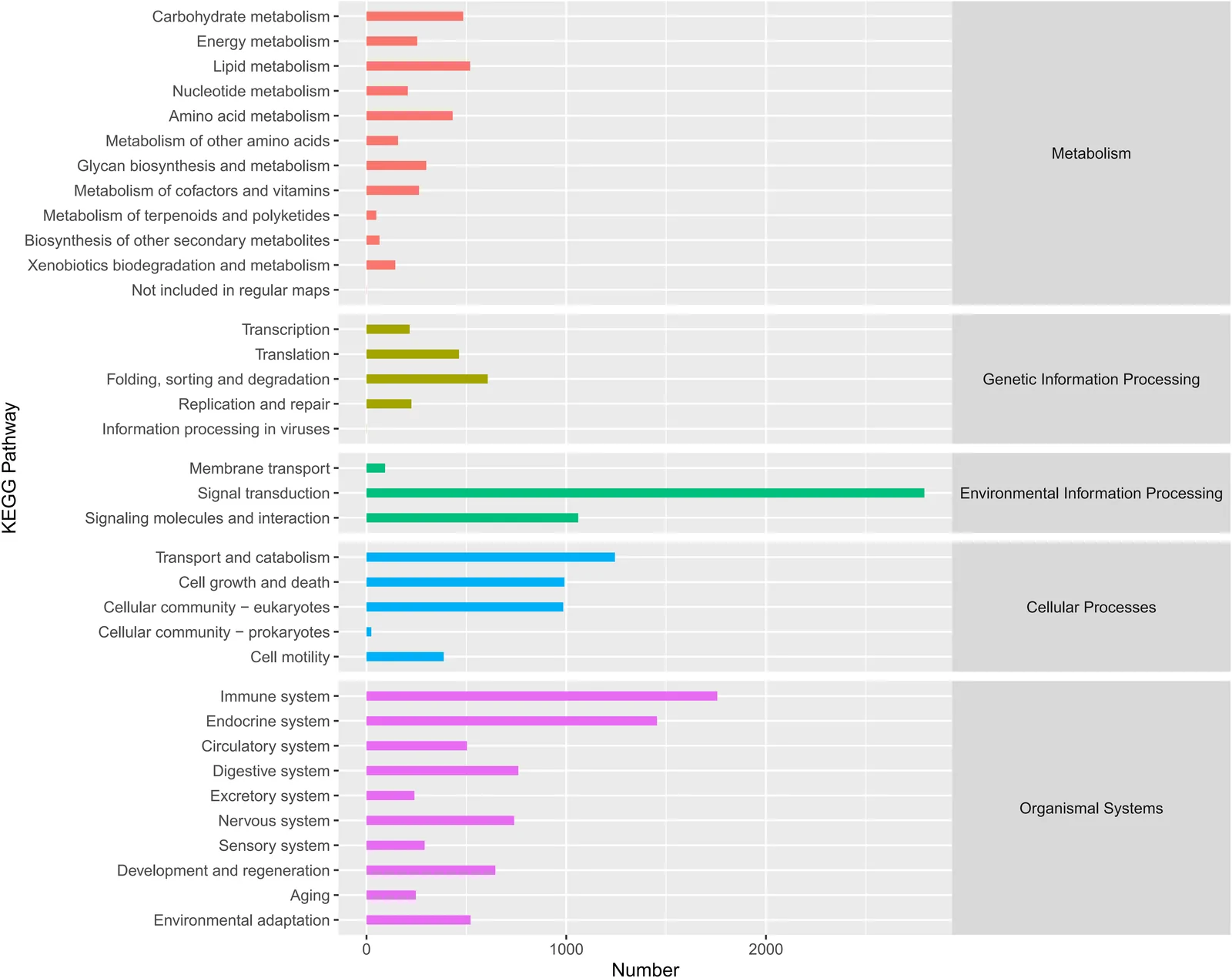
KEGG annotation statistics.

#### 3.2.3 GO annotation

According to the annotation results from the GO database (**Figure 4**), the Unigenes were annotated to three ontologies of Gene Ontology (GO), which describe the molecular function, cellular component, and biological process of genes. The statistical results show that the number of Unigenes annotated to the biological process category is 3,125,852, the number of those annotated to the molecular function category is 3,201,070, and the number of those annotated to the cellular component category is 3,201,089.

**Figure 4:**
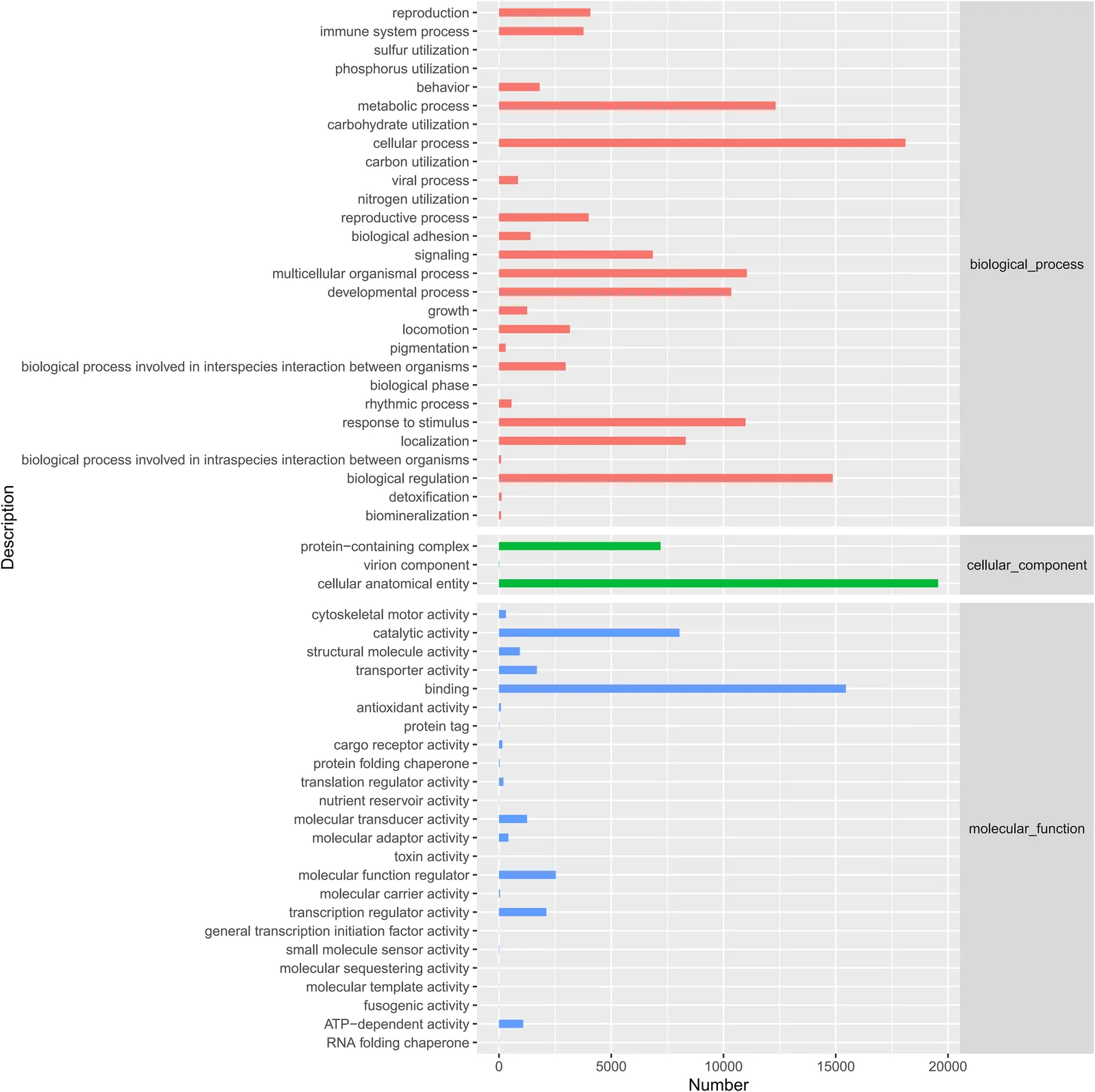
GO secondary classification statistical chart. The results are summarized into three ontologies (Cellular Component, Biological Function and Molecular Function).

Among the biological process category, the three secondary classifications with the highest annotation numbers are biochemical process (19,307), cell protrusion (18,587), and biological regulation (14,871). In the molecular function category, the top three secondary classifications with the annotation numbers are binding (15,453), catalytic activity (8,042), and hydrolase activity (3,633). In the cellular component category, the three secondary classifications with the highest annotation numbers are cell anatomical entity (19,476), intracellular organelle (15,172), and cytoplasm (14,815).

### 3.3 Differential expression analysis

#### 3.3.1 Overall result of expression difference analysis

The DESeq software was used to conduct differential analysis of gene expression. The screening conditions for differentially expressed genes were: the fold change in expression | log2FoldChange | > 1, and the significance P-value < 0.05. The analysis was performed on the differentially expressed genes in the CK1 group and the B2 group. The results showed that compared with the CK1 group, a total of 1,080 differentially expressed genes were identified in the B2 group, among which 509 genes had upregulated expression and 571 genes had downregulated expression (**Figure 5**).

**Figure 5:**
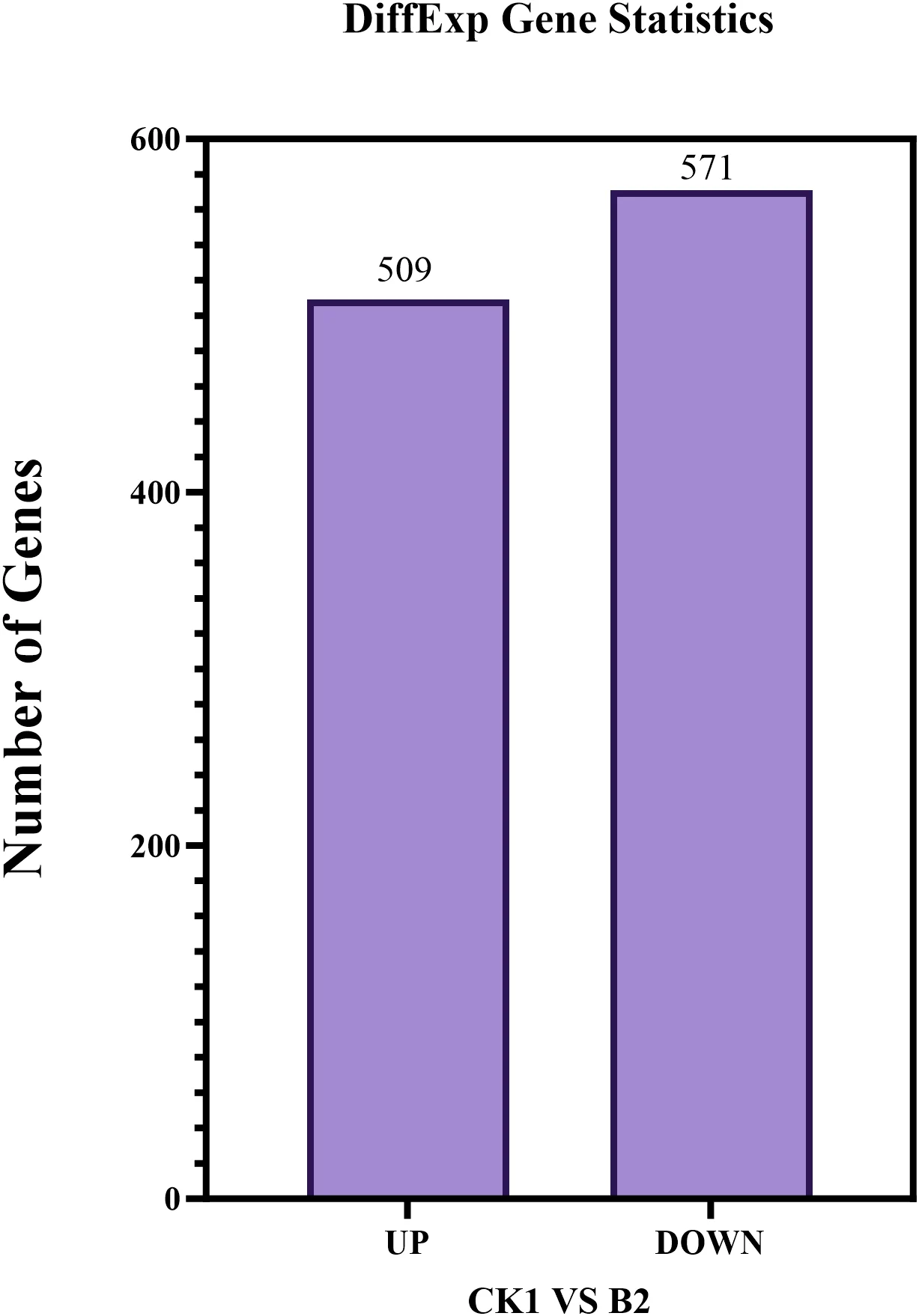
Differential gene statistical map. Abscissa: samples compared in pairs; Ordinate: the number of differentially expressed genes.

To visually display the differences in gene expression between the two groups, a volcano plot was drawn. The volcano plot can clearly reflect the significant differences in gene expression between the CK1 group and the B2 group. The farther the gene point is from the center, the greater the degree of expression difference (**Figure 6**).

**Figure 6:**
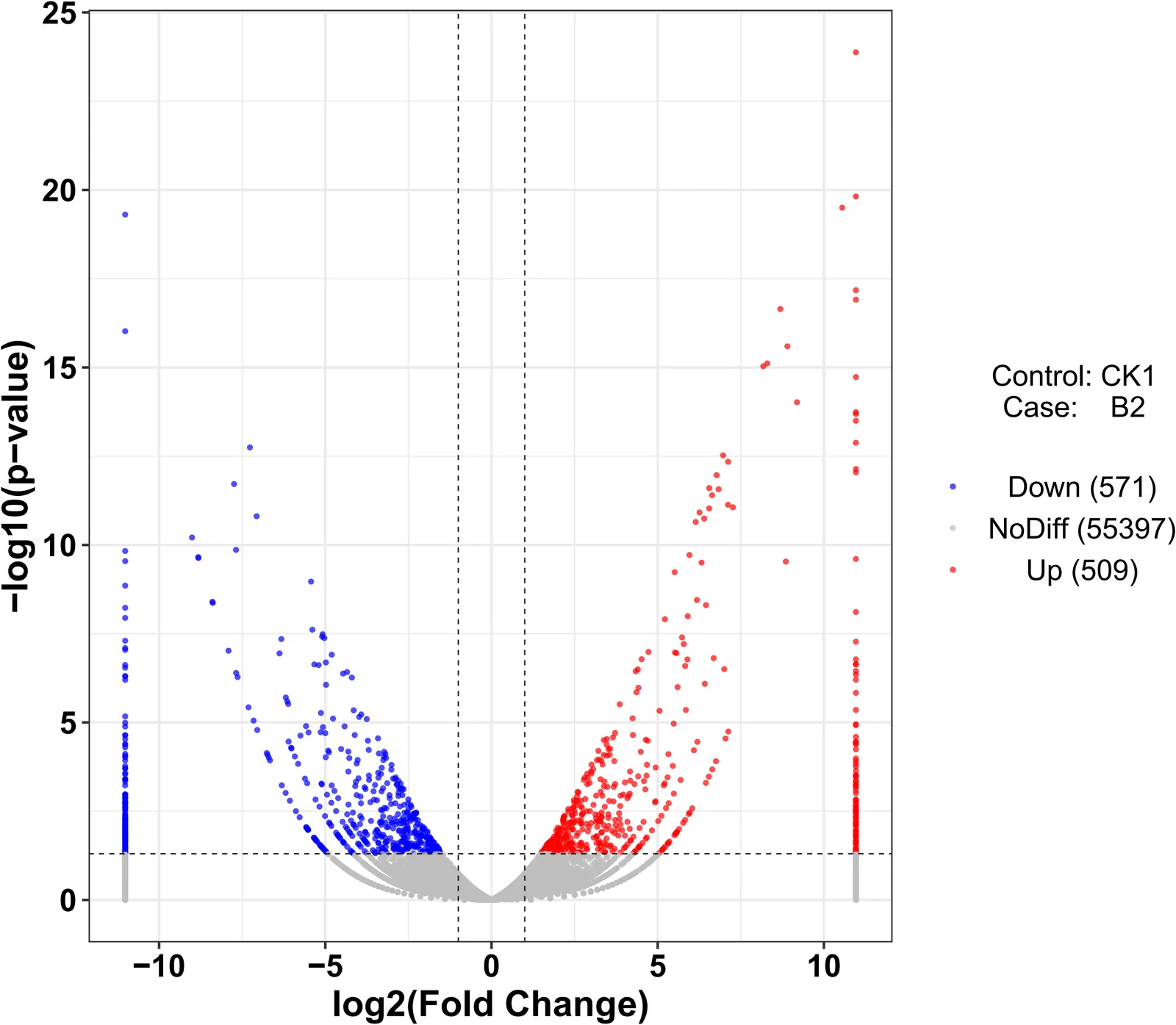
Differential comparison volcano map. The abscissa represents the logarithmic value of the difference multiple between the two groups, and the ordinate represents the negative Log10 value of the difference FDR between the two groups. The red (up-regulated expression of Group B2 relative to Group CK1) and blue (down-regulated expression) dots indicate that the gene expression is different (the judging standard is FDR <0.05, and the difference multiple is more than twice), and the gray dots indicate that there is no difference.

#### 3.3.2 GO enrichment analysis

The GO enrichment analysis was conducted using the topGO software. Based on the GO term annotation information, the number of differential genes and the gene list in each term were calculated. The P-value (significance threshold set as P-value < 0.05) was calculated through the hypergeometric distribution test to identify the GO terms that significantly enriched the differential genes compared to the entire genomic background. This allowed for the interpretation of the main biological functions that the differential genes were involved in. The GO enrichment results of the differentially expressed genes were classified according to biological process (BP), cellular component (CC), and molecular function (MF), and the top 10 GO terms with the most significant enrichment (lowest P-value) in each category were selected for presentation. The results are shown in **Figure 7**. The analysis results indicated that the differential genes were significantly enriched in the three functional categories of biological process, cellular component, and molecular function. In the biological process category, the differential genes were mainly enriched in items such as cell process, cell protrusion, metabolic process, biological regulation, multicellular organism process, biochemical process, regulation of biological process, and stress response; in the cellular component category, the differential genes were mainly distributed in cellular anatomical entities, intracellular organelles, and protein-containing complexes; in the molecular function category, the differential genes were mainly involved in functions such as binding, catalytic activity, and hydrolase activity.

**Figure 7:**
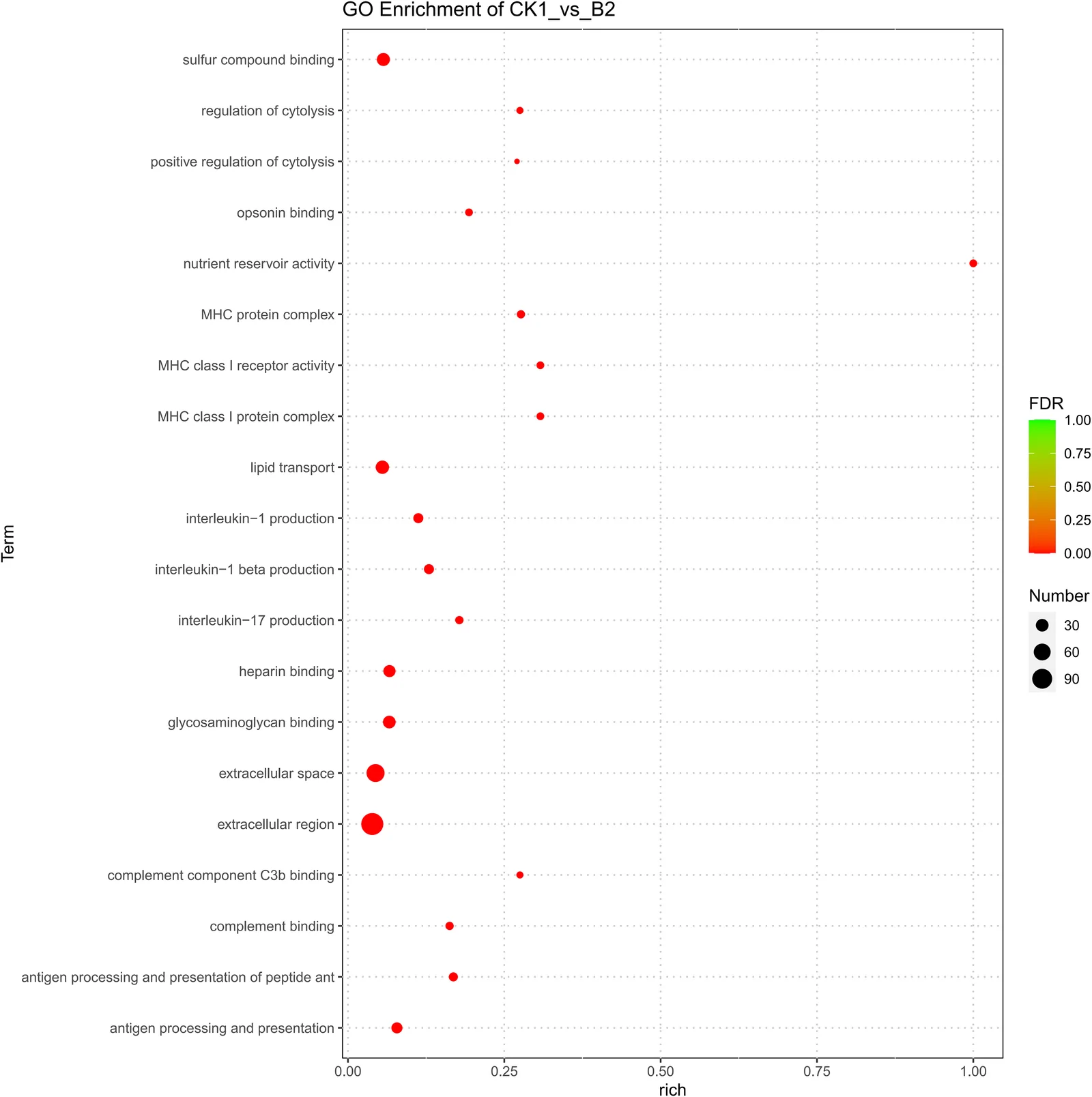
Column chart of GO enrichment classification. The abscissa is the secondary GOterm, and the ordinate is the number of differential genes in this term.

#### 3.3.3 KEGG enrichment analysis

Through KEGG pathway enrichment analysis, the differentially expressed genes were enriched in a total of 281 pathways. In the KEGG enrichment analysis, the significance judgment criteria usually include P-value < 0.05 or the corrected P-value (such as FDR) < 0.05. In the bar chart, the enrichment significance is represented by -log10(P-value), and when this value is greater than 1.3 (corresponding to P-value < 0.05), it is considered as significant enrichment. Based on the KEGG enrichment analysis results of the differentially expressed genes, the top 30 pathways with the most significant enrichment (the smallest P-value) were selected for presentation. The results are shown in **Figure 8**. As shown in the figure, the enrichment significance of the first-level classifications such as Cellular Processes, Environmental Information Processing, and Genetic Information Processing is much higher than the threshold, indicating that in the B2 treatment group, the related pathways under these classification categories have significant enrichment. Specifically, the significantly enriched pathways mainly involve human diseases (such as autoimmune thyroid disease, type 1 diabetes, graft-versus-host disease, etc.), biological systems (such as antigen processing and presentation, pancreatic juice secretion, complement and coagulation cascade reaction, etc.), cell processes (such as phagosome), and environmental information processing (such as cell adhesion molecules), suggesting that feeding the *S. cathayensis* feed may play an important role in improving the health status of loaches by regulating these pathways.

**Figure 8:**
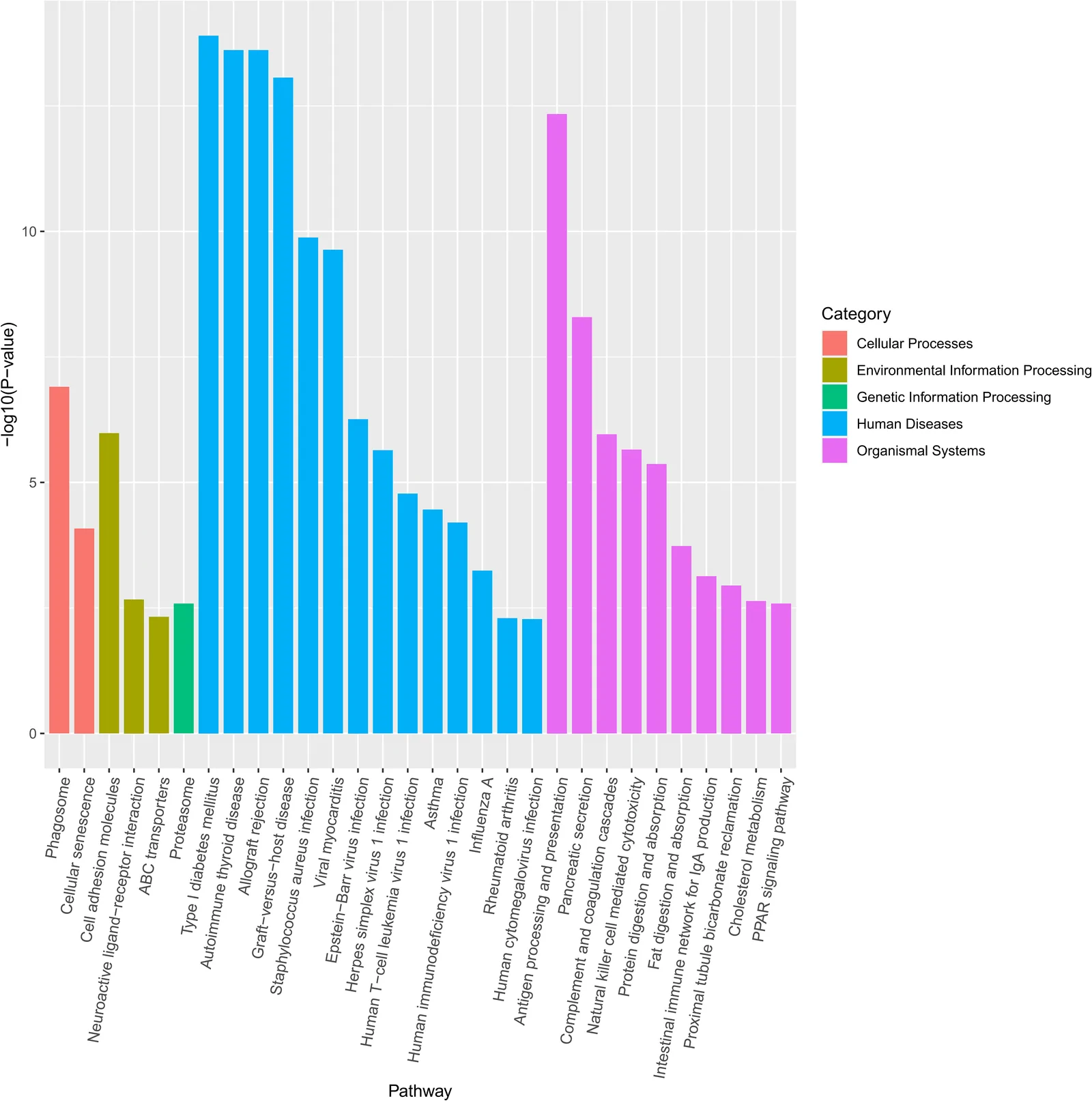
KEGG enrichment bar chart. The top 30 Pathways with the smallest p-values are selected for display. The x-axis represents the pathway names, and the y-axis represents the -log10(p-value) of enrichment for each pathway.

### 3.4 qRT-PCR validation of DEG expression levels

To verify the accuracy of the RNA-seq sequencing results, this study employed qRT-PCR technology to validate the expression levels of 12 differentially expressed genes in the B2 treatment group. The results showed that the expression trends of all the validated genes were consistent with the RNA-seq sequencing results. Among them, 7 genes showed downregulation, and 5 genes showed upregulation, and the differences were statistically significant, indicating that the RNA-seq data in this study had high reliability (**Figure 9**).

**Figure 9:**
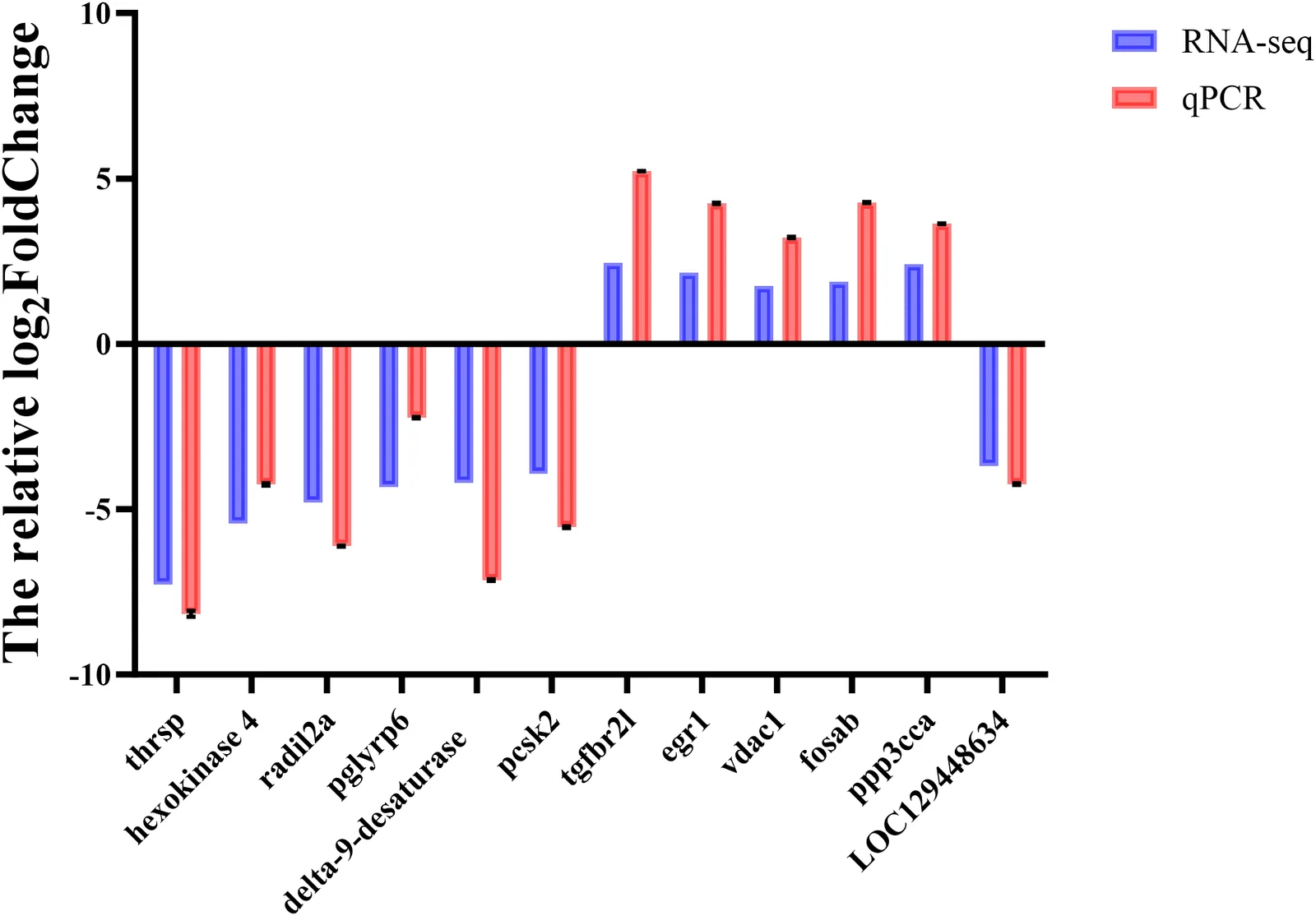
qRT-PCR validation of RNA-seq results. The relative log2FoldChange is shown by the ordinate, and the gene is represented by the abscissa. Genes that are up-regulated are above the horizontal line, while those that are down-regulated are below it.

### 3.5 Effects of *Semiliquidambar cathayensis* extract supplementation on intestinal microbial composition

The dominant species are the key factors determining the ecological functions and structural characteristics of microbial communities. Therefore, analyzing the species composition of communities at different taxonomic levels helps to deeply understand the construction process, dynamic changes, and ecological significance of community structures. This study conducted statistical analysis on the species composition of each sample at various taxonomic levels and presented the changes in species abundance among different samples in a stacked graph format. **Figure 10** shows the composition of taxa at different taxonomic levels, including phyla (**Figure 10**), classes (**Figure 10**), orders (**Figure 10**), families (**Figure 10**), genera (**Figure 10**), and species (**Figure 1**). The top 10 species with the highest average abundance in all samples were selected for presentation. At the phylum level, the dominant phyla in the CK1 group were Bacteroidetes (71.06%) and Proteobacteria (28.76%), while in the B2 group, the dominant phylum was Bacteroidetes (80.52%), followed by Proteobacteria (17.54%); at the class level, the dominant class in the CK1 group was Flavobacteriia (39.09%), followed by Bacillia (31.41%) and γ-Proteobacteria (20.27%), and in the B2 group, the dominant class was Flavobacteriia (61.92%), followed by Bacillia (17.91%) and γ-Proteobacteria (14.92%); at the order level, the dominant order in the CK1 group was Flavobacteniales (39.09%) and Lactobacillales (31.41%), while in the B2 group, the dominant order was Flavobacteniales (61.92%) and Lactobacillales (17.91%); at the family level, the dominant family in the CK1 group was Streptococcaceae (31.07%), followed by Flavobacteniaceae (20.48%) and Weeksellaceae (18.61%), and in the B2 group, the dominant family was Weeksellaceae (61.80%) and Streptococcaceae (17.88%); at the genus level, the dominant genus in the CK1 group was Lactococcus (31.02%), Myroides (19.90%), and Elizabethkingia (11.87%), while in the B2 group, the dominant genus was Elizabethkingia (61.70%) and Lactococcus (17.60%); at the species level, the dominant species in the CK1 group was unclassified species, accounting for 63.77%, while in the B2 group, the dominant species was Elizabethkingia meningoseptica (61.70%). In addition, the main species in the CK1 group were Mvroidesodoratimimus (19.90%) and Elizabethkingia meningoseptica (11.87%).

**Figure 10:**
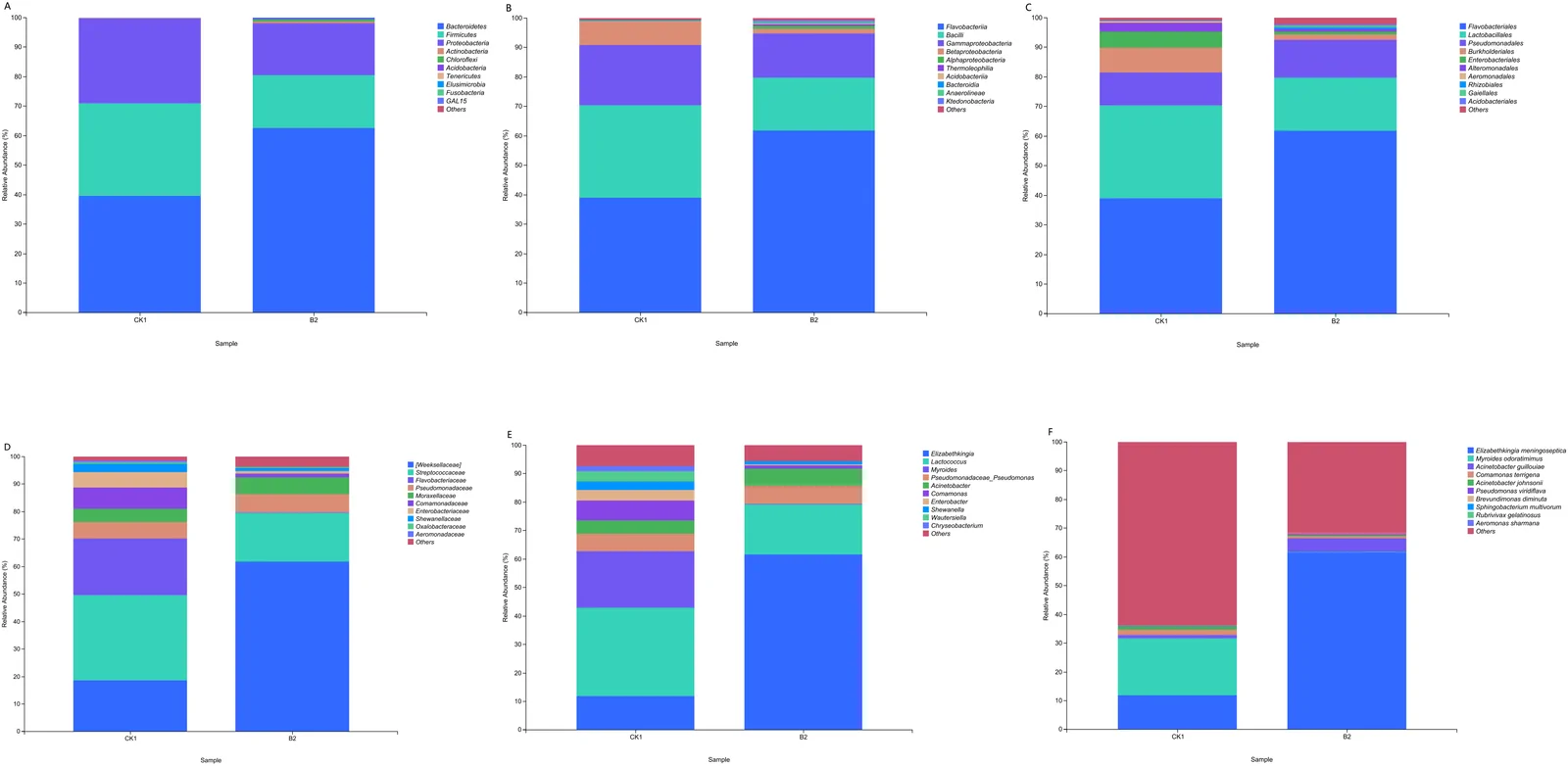
The effect of adding *Semiliquidambar cathayensis* oil ether extract to the feed on the relative abundance of intestinal microbial communities at different classification levels. **(A)** phylum, **(B)** class, **(C)** order, **(D)** family, **(E)** genus, **(F)** species.

To identify potential biomarkers in the intestinal microbiota of *M. anguillicaudatus* after *A. hydrophila* infection, this study constructed a Venn diagram based on the abundance data of amplicon sequence variants and operational taxonomic units to compare the common and unique species compositions between the CK1 and B2 sample groups. The results showed that a total of 386 operational taxonomic units were detected in the intestinal microbiota of the two groups of mudfish. Among them, 71 operational taxonomic units were common to both the CK1 and B2 groups, 77 operational taxonomic units were unique to the CK1 group, and 238 operational taxonomic units were unique to the B2 group (**Figure 11**).

**Figure 11:**
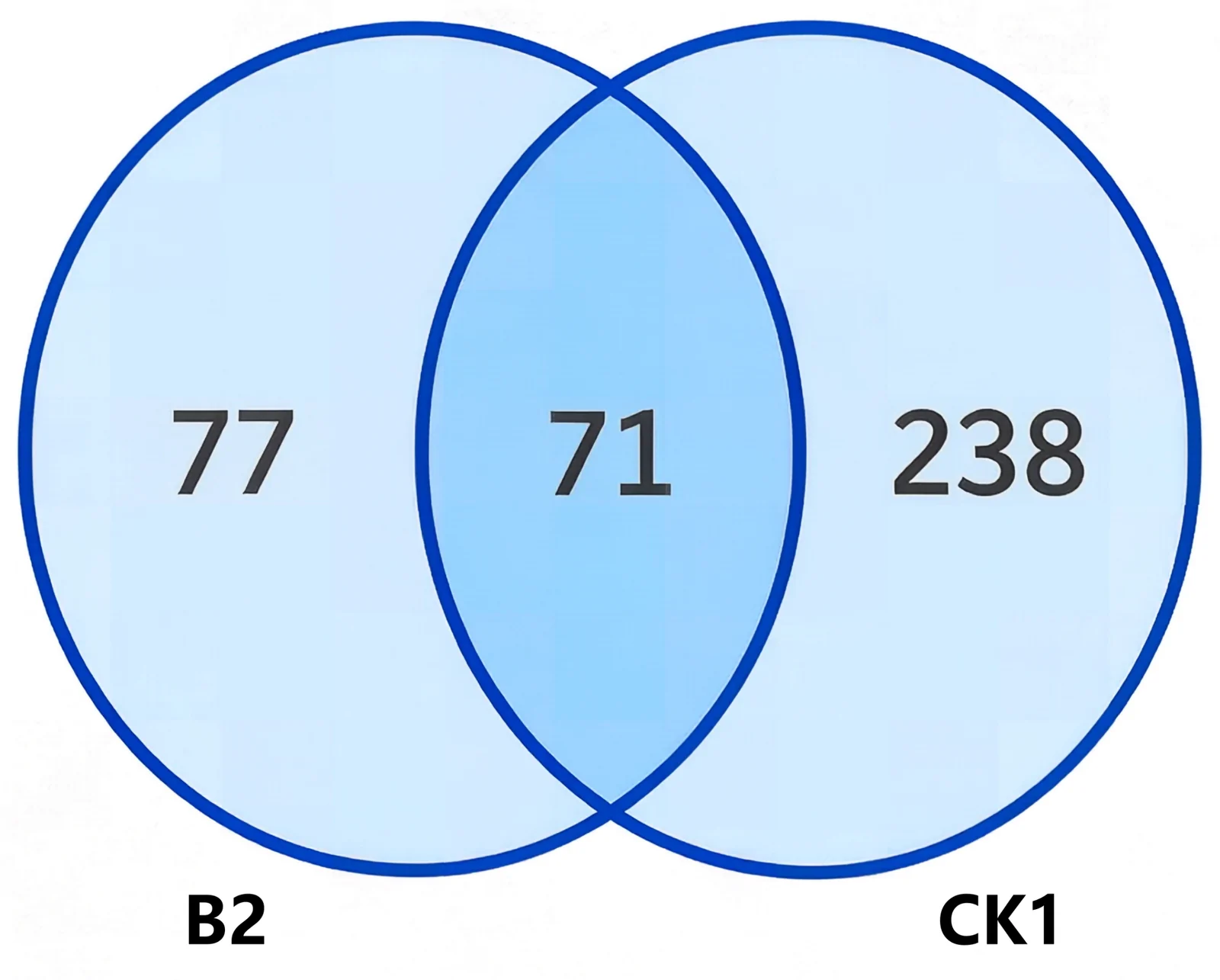
Different species and marker species analysis through Venn diagram in *Misgurnus anguillicaudatus* microbiota induced by *Aeromonas hydrophila*. Groups CK1 and B2 in the figure represent the control and *Semiliquidambar cathayensis* treatment, respectively.

### 3.6 Effects of adding *Semiliquidambar cathayensis* on the alpha diversity

**Table 5** presents the statistical analysis results of various α diversity indices such as Chao1 index, Faith_PD index, Good coverage index, Observed species index, Pielou evenness index, Shannon index, and Simpson index. The Good coverage index of both sample groups is at a relatively high level, indicating that the sequencing data volume is sufficient. In terms of other diversity indices, the values of the CK1 group are all higher than those of the B2 group, suggesting that the gut microbiota in the CK1 group is superior to that in the B2 group in terms of species richness, evenness, and phylogenetic diversity. *S. cathayensis* may to some extent alleviate the damage to the diversity of the microbiota. The sequencing quality assessment results show that the Simpson index of both sample groups in the dilution curve (**Supplementary Figure 1**) has tended to plateau, indicating that the sequencing depth is sufficient to cover the vast majority of species in the samples; the rank abundance curve (**Supplementary Figure 1**) shows a gentle trend in the vertical direction, reflecting a relatively uniform distribution of the abundance of operational taxonomic units in the samples.

**Table 5:** Statistical analysis of index in alpha diversity.

Index	Chao1	Faith_ PD	Goods_Coverage	Observed _species	Pielou	Shannon	Simpson
CK1	325.906	15.5865	0.998295	306.2	0.592697	4.89466	0.887713
B2	157.493	11.7025	0.999407	148.4	0.367138	2.64825	0.601183

Chao1, Faith_ PD, Goods_Coverage, Observed _species, Pielou, Shannon and Simpson, which is mainstream alpha diversity index and their correlation analysis results.

### 3.7 Species differences and analysis of indicator species

To compare the differences in species composition among the samples, a heatmap was drawn based on the top 20 genera in terms of average abundance. The heatmap could be sorted by sample order or clustering results, and the analysis and visualization were completed using the pheatmap package in R language. See **Figure 12**. Based on the genus-level species composition shown in the heatmap, a comparative analysis of the intestinal microbiome structure of the control group (CK1) and the treatment group (B2) was conducted. The results showed that Weeksella had a higher abundance in the CK1 group, while the relative abundances of pathogenic conditions-causing genera such as Aeromonas, Shewanella, and Klebsiella in the B2 group significantly increased. At the same time, the abundances of genera such as Cloacibacterium, Comamonas, and Chryseobacterium in the B2 group also changed significantly, indicating that the infection of *A. hydrophila* led to the remodeling of the intestinal microbiome structure, and the abundance ratio of beneficial bacteria to pathogenic conditions-causing bacteria was out of balance. The above-differentiated species may serve as key biomarkers for distinguishing the two sample groups and participate in regulating the host immune response process. In addition, PCoA is based on the dimensionality reduction of the distance matrix and is more suitable for microbial data with a large number of species and a long-tailed distribution of abundances; for samples with a simpler species composition, PCA can be used to linearly combine the species difference features into principal components, and the differences between samples can be quantified based on the scores. The above analysis was implemented using R language. See **Supplementary Figure 2**. Based on the PCoA analysis results, the control group (CK1) and the treatment group (B2) showed a significant separation on the PCo1 axis, with the explanatory degree of PCo1 for sample differences being 81.7%, indicating that there were significant differences in the intestinal microbiome structure between the two groups. The samples in the CK1 group clustered in the positive direction of PCo1, while those in the B2 group shifted toward the negative direction, suggesting that the infection of *A. hydrophila* induced a directional change in the composition of the intestinal microbiome. The explanatory degree of PCo2 was 10.3%, further reflecting some of the internal variations in the two sample groups. Overall, the PCoA sorting diagram clearly shows the differences in the microbiome structure between the CK1 and B2 sample groups.

**Figure 12:**
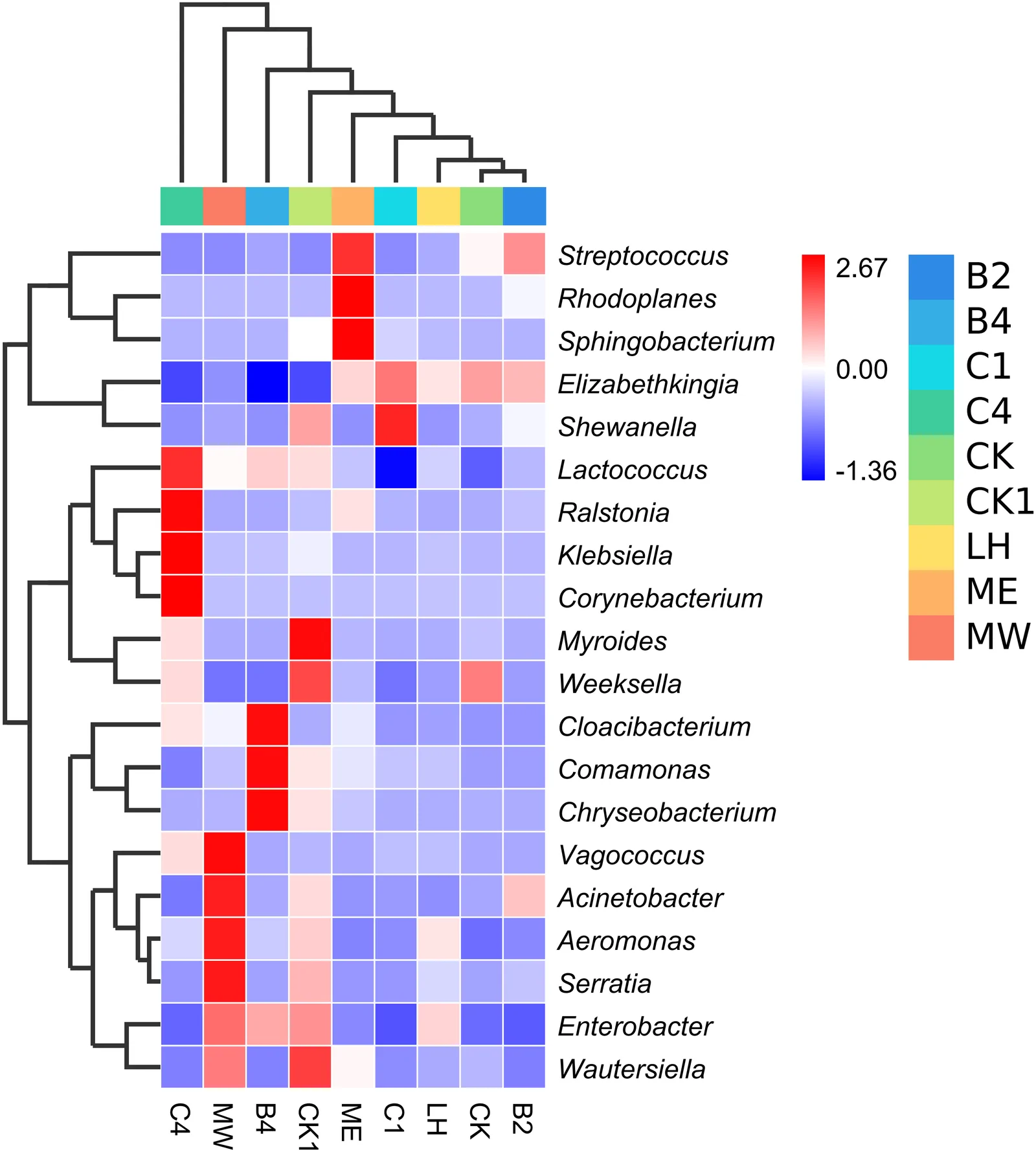
Species composition heatmap. Based on the heatmap displaying genus-level species composition, the intestinal microbiota structure was compared between the control group (CK1) and the treatment group (B2). The rows and columns of the heatmap were ordered according to the clustering tree derived from inter-sample correlations.

### 3.8 Based on functional potential prediction

Based on the functional prediction results, the abundance values of each functional unit can be calculated using the metabolic pathway database. Commonly used databases include: the KEGG database, whose core is metabolic pathway analysis, which divides the pathways into six categories such as metabolism and genetic information processing, and further subdivides them into multiple levels; the MetaCyc database, which includes primary and secondary metabolic pathways and related enzyme and gene information verified through experiments; the COG database, which is used for homologous protein function annotation. The analysis is conducted using R language, based on the normalized pathway abundance table, to calculate the average abundance or total abundance of each sample in the second-level pathways. Based on the KEGG, MetaCyc and COG databases, the normalized abundance table is calculated using R language to obtain the average abundance or total abundance of the second-level metabolic pathways. As can be seen from **Figure 13**, the pathways with higher abundance are mainly concentrated in basic metabolic categories such as carbohydrate metabolism, amino acid metabolism, and energy metabolism, indicating that the gut microbiota plays an important role in the host’s energy and nutrient metabolism. In addition, some pathways related to signal transduction, membrane transport and environmental adaptation also show high abundance levels, reflecting the close interaction between the microbial community and the host environment. Therefore, further analysis of the differences in the species composition level of the differential microbiota shows that there are significant differences in the genus level between the CK1 group and the B2 group (**Figure 14**). The dominant genus in the CK1 group is Lactococcus, while in the B2 group, Elizabethkingia is in absolute dominance, with a significantly higher abundance than the CK1 group. In addition, genera such as Pseudomonas, Comamonas, and Myroides also show different distribution characteristics between the two groups. Based on the functional prediction results, the significant increase in the abundance of Elizabethkingia in the B2 group may be closely related to the enrichment of pathways such as carbohydrate metabolism and amino acid metabolism, suggesting that the treatment of *S. cathayensis* may regulate the specific microbial community structure and thereby affect the metabolic function of the gut microbiota (**Figure 15**).

**Figure 13:**
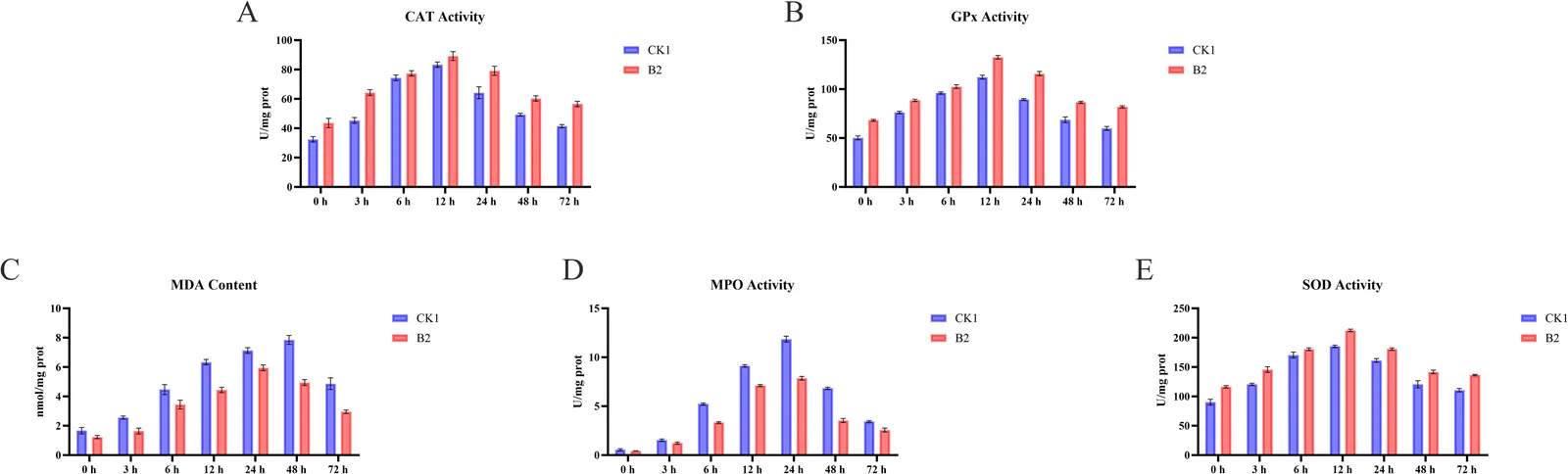
Analysis of physiological indicator results: **(A)** Superoxide Dismutase (SOD) experiment, **(B)** Catalase (CAT) experiment, **(C)** Glutathione Peroxidase (GPx) experiment, **(D)** Malondialdehyde (MDA) experiment, **(E)** Myeloperoxidase (MPO) experiment.

**Figure 14:**
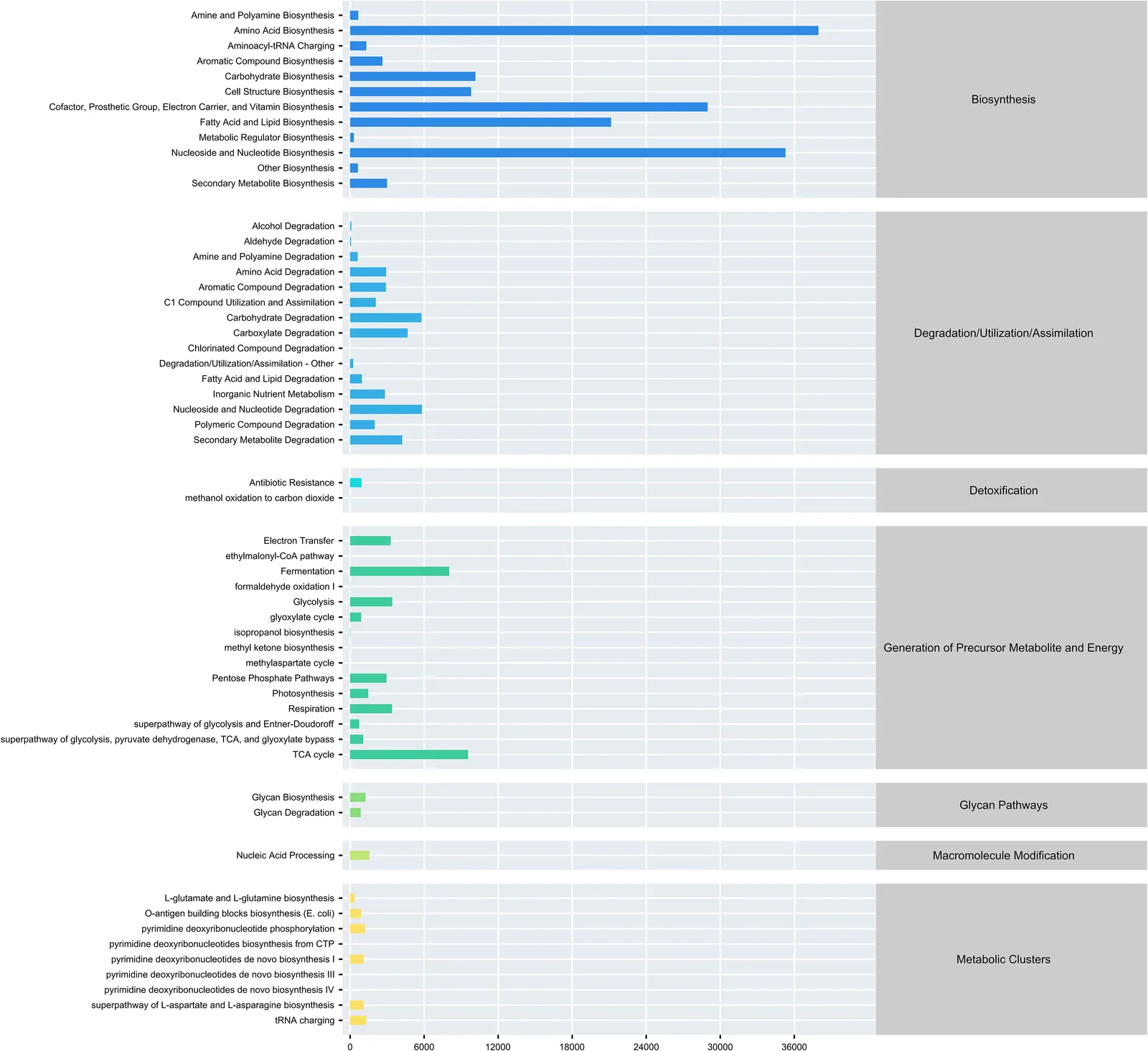
Statistics of metabolic pathways.

**Figure 15:**
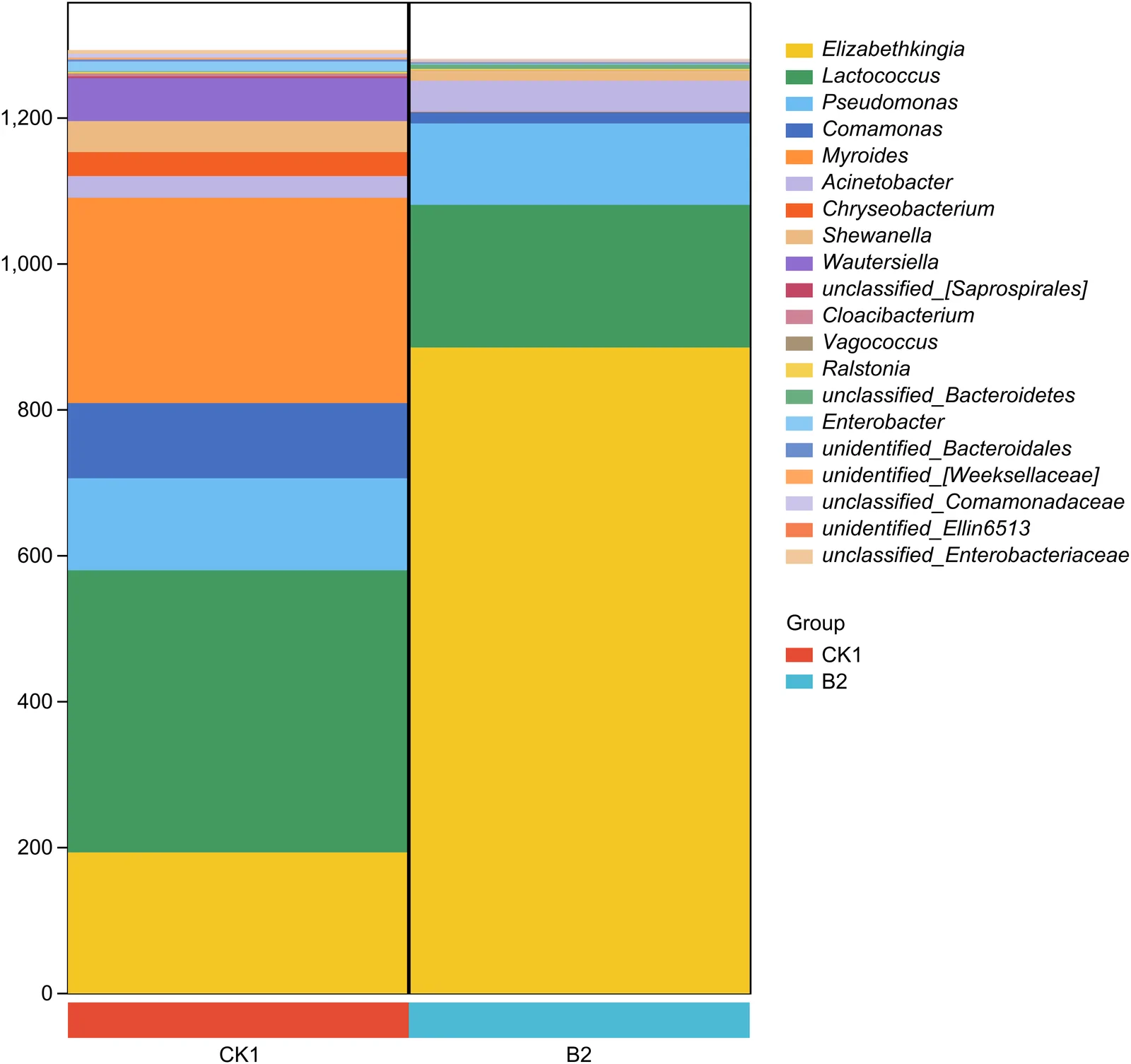
Differential analysis of metabolic pathways.

### 3.9 Analysis of physiological indicators results

Based on the results of liver tissue physiological indicators (**Figure 13**), this study systematically explored the regulatory effect of the petroleum ether extract of *S. cathayensis* (B2) on liver oxidative stress and inflammatory response in *M. anguillicaudatus* infected with *A. hydrophila*. The results showed that B2 could significantly enhance the liver’s antioxidant capacity and alleviate liver damage and inflammation caused by infection. The activities of superoxide dismutase (SOD), catalase (CAT), and glutathione peroxidase (GPx) in the B2 group were significantly increased and maintained at a high level from 24 to 72 hours after infection, suggesting that it could enhance the body’s ability to clear free radicals. At the same time, B2 significantly reduced the content of malondialdehyde (MDA), a terminal product of lipid peroxidation, and alleviated oxidative damage to liver cell membranes. In addition, B2 significantly inhibited the activity of myeloperoxidase (MPO), a marker enzyme of neutrophils, and effectively alleviated neutrophil infiltration and inflammatory response in the liver from 12 to 48 hours after infection. This indicates that B2 can protect the liver of *M. anguillicaudatus* from damage caused by *A. hydrophila* infection by enhancing antioxidant capacity, inhibiting lipid peroxidation and inflammatory infiltration.

## 4 Discussion

This study systematically explored the regulatory mechanism of the extract of *S. cathayensis* on the immune stress induced by *A. hydrophila* in common carp by integrating 16S rRNA sequencing and transcriptomics techniques. The results showed that the treatment with *S. cathayensis* significantly reshaped the intestinal microbiota structure of common carp and regulated the expression of host immune-related genes, suggesting that it may exert immune regulatory effects through the “microbiota-host” interaction network.

At the host transcriptome level, 1,080 differentially expressed genes were identified after treatment with *S. cathayensis*. GO enrichment analysis showed that these genes were mainly involved in cellular processes, biological regulation, and stress responses. KEGG enrichment analysis revealed that they were significantly enriched in immune-related pathways such as antigen processing and presentation, complement and coagulation cascade reactions, and phagosome, indicating that Semiliquidambar cathayensis can enhance the host’s immune defense capacity by regulating these pathways. Additionally, the enrichment of disease-related pathways such as autoimmune thyroid diseases and type 1 diabetes further supports the immunomodulatory effect of *S. cathayensis*. The composition of the host’s gut microbiota is shaped by various factors, and in turn, the microbiota regulates multiple physiological functions of the host, including nutrient digestion and absorption, immune modulation, and defense against pathogenic bacteria (22–24). At the gut microbiota level, treatment with *S. cathayensis* led to a significant decrease in the α-diversity index of the microbiota, with the dominant genera shifting from Lactococcus and Myroides to Elizabethkingia, with Elizabethkingia meningoseptica accounting for an absolute majority in the B2 group, reaching up to 61.70%. PCoA analysis further confirmed the significant separation of the microbiota structures between the two groups, indicating that *S. cathayensis* induced an overall remodeling of the gut microbiota. This change may be related to the targeted regulation of specific microbiota by flavonoids and phenolic acids in *S. cathayensis*. The integrated analysis showed that the significantly enriched basic metabolic pathways such as carbohydrate metabolism and amino acid metabolism in the B2 group were highly consistent with the functional characteristics of the dominant genus Elizabethkingia, which has the ability to metabolize complex polysaccharides and participate in the nitrogen cycle, and may regulate host immune gene expression by producing short-chain fatty acids and other metabolites. At the same time, the decrease in the abundance of Lactococcus in the B2 group was potentially associated with the differential expression of host immune-related genes, reflecting the negative selection of the host immune state on specific microbiota. The present study demonstrated that *S. cathayensis* petroleum ether extract (B2) effectively alleviated hepatic oxidative damage and inflammatory infiltration induced by *A. hydrophila* in *M. anguillicaudatus*, primarily by enhancing antioxidant enzyme activities, inhibiting lipid peroxidation, and suppressing myeloperoxidase-mediated inflammation.

In conclusion, *S. cathayensis* may exert its immunomodulatory effect by regulating the structure of the intestinal microbiota, specifically increasing the abundance of certain bacterial groups such as Elizabethella. This, in turn, affects the expression of host immune-related genes, thereby alleviating the immune stress induced by *A. hydrophila*. This study provides a theoretical basis for the development and application of *S. cathayensis* as an aquatic immune enhancer, and also offers a new research perspective for exploring the immune regulatory role of traditional Chinese medicine through the regulation of “microbiota-host” interactions.

## 5 Conclusion

This study systematically explored the regulatory mechanism of the extract of *S. cathayensis* on the immune stress induced by *A. hydrophila* in *M. anguillicaudatus* through integrating transcriptomics and 16S rRNA sequencing technology. The transcriptomic analysis revealed that a total of 1,080 differentially expressed genes were screened out after treatment with *S. cathayensis*, mainly enriched in immune-related pathways such as antigen processing and presentation, complement and coagulation cascade reactions, and phagosome. This indicates that *S. cathayensis* can regulate the expression of host immune genes. The 16S rRNA sequencing results showed that the treatment with *S. cathayensis* significantly reshaped the intestinal microbiota structure, with the dominant genera shifting from Lactococcus and Myroides to Elizabethkingia, which was in absolute dominance. Furthermore, the research results indicate that the extract of *S. cathayensis* can protect the liver of *M. anguillicaudatus* from damage caused by *A. hydrophila* by enhancing antioxidant capacity, reducing oxidative stress and inflammatory responses. The integrated analysis revealed that *S. cathayensis* may affect the expression of host immune-related genes by regulating the abundance of specific microbiota (especially Elizabethkingia), thereby alleviating the immune stress induced by *A. hydrophila*. This study provides a theoretical basis for the development and application of *S. cathayensis* as an aquatic immune enhancer, and also offers a new research perspective for exploring the immune regulatory role of traditional Chinese medicine through “microbiota-host” interaction.

## Data Availability

The datasets presented in this study can be found in online repositories. The names of the repository/repositories and accession number(s) can be found below: https://www.ncbi.nlm.nih.gov/genbank/, SRR29791122.

## Supplementary material

Supplementary Figure 1 Alpha-diversity index analysis. **(A)** Rarefaction curve, **(B)** RankAbundance. Groups CK1 and B2 in the figure represent the control and *Semiliquidambar cathayensis* treatment, respectively.

Supplementary Figure 2 PCA analysis. By ranking the samples based on their scores on these principal component indicators, the degree of difference in species composition among samples can be quantitatively visualized.
